# Identification of the Signature Genes and Network of Reactive Oxygen Species Related Genes and DNA Repair Genes in Lung Adenocarcinoma

**DOI:** 10.3389/fmed.2022.833829

**Published:** 2022-02-21

**Authors:** Ye Zhao, Hai-Ming Feng, Wei-Jian Yan, Yu Qin

**Affiliations:** ^1^First Clinical Medical College, Lanzhou University, Lanzhou, China; ^2^Department of Thoracic Surgery, The Second Affiliated Hospital of Lanzhou University, Lanzhou, China

**Keywords:** DNA repair, lung adenocarcinoma, prognostic analysis, reactive oxygen species (ROS), regulatory network

## Abstract

Reactive Oxygen Species (ROS) are present in excess amounts in patients with tumors, and these ROS can kill and destroy tumor cells. Therefore, tumor cells upregulate ROS-related genes to protect them and reduce their destructing effects. Cancer cells already damaged by ROS can be repaired by expressing DNA repair genes consequently promoting their proliferation. The present study aimed to identify the signature genes of and regulating network of ROS-related genes and DNA repair genes in lung adenocarcinoma (LUAD) using transcriptomic data of public databases. The LUAD transcriptome data in the TCGA database and gene expressions from Gene Expression Omnibus (GEO) were analyzed and samples were clustered into 5 ROS-related categories and 6 DNA repair categories. Survival analysis revealed a significant difference in patient survival between the two classification methods. In addition, the samples corresponding to the two categories overlap, thus, the gene expression profile of the same sample with different categories and survival prognosis was further explored, and the connection between ROS-related and DNA repair genes was investigated. The interactive sample recombination classification was used, revealing that the patient's prognosis was worse when the ROS-related and DNA repair genes were expressed at the same time. The further research on the potential regulatory network of the two categories of genes and the correlation analysis revealed that ROS-related genes and DNA repair genes have a mutual regulatory relationship. The ROS-related genes namely NQO1, TXNRD1, and PRDX4 could establish links with other DNA repair genes through the DNA repair gene NEIL3, thereby balancing the level of ROS. Therefore, targeting ROS-related genes and DNA repair genes might be a promising strategy in the treatment of LUAD. Finally, a survival prognostic model of ROS-related genes and DNA repair genes was established (TERT, PRKDC, PTTG1, SMUG1, TXNRD1, CAT, H2AFX, and PFKP). The risk score obtained from our survival prognostic model could be used as an independent prognostic factor in LUAD patients.

## Introduction

Reactive oxygen species (ROS) are small oxygen-derived active small molecules, including O2·-,·OH, RO2·, and RO·(1). ROS can be produced by exogenous or endogenous sources, and when they are in excess amount, compared with the concentration of antioxidants in the body, the system is out of balance, and the antioxidants are not able anymore to completely remove or reduce ROS. On the one hand, their accumulation damages biological macromolecules, including DNA, leading to different type of tumors. On the other hand, the increase of the level of intracellular ROS can allow the selective killing of tumor cells (2). A high ROS amount is detected in most cancer patients (3). The expression of ROS-related proteins increases in many types of cancer, and they are involved in cell growth, proliferation, differentiation, protein synthesis, glucose metabolism, cell survival and inflammation (4). Oxidative stress and non-small cell lung cancer (NSCLC) have a mutually promoting and dependent relationship (5–9). Indeed, the presence of oxidative stress greatly increases gene damage, and the damage to the mitochondrial DNA of alveolar cells can cause energy supply barriers, promote tumor blood vessel formation, and inhibit tumor immune microenvironment. These multiple effects promote the occurrence of NSCLC. In addition, the abnormal expression of specific transcription factors and downstream cell signaling pathways caused by and related to oxidative stress allow a rapid development and metastasis of NSCLC. Furthermore, NSCLC cells maintain the oxidative stress response at the appropriate level for their proliferation and survival by regulating their antioxidant levels and ROS levels (10, 11).

The internal and external environmental factors including ROS can cause DNA damage. If the damage is not repaired in time and correctly, it causes the instability of the genome, threatening the survival of cells. In order to maintain the stability of the structure and function of DNA in a complex genomic environment, a timely and reasonable response to damaged signals should be provided. Under the condition of DNA damage, coordinated regulation of damage repair mechanisms and dynamic chromatin changes are required for the maintenance of genetic and epigenetic information. Thus, cells should correct the damages before the replication process in order to maintain the integrity of the genetic material. Therefore, the DNA repair system plays a vital role in maintaining the normal physiological functions of cells (12). At present, more than 100 repair enzymes are known that participate in the DNA repair process. The DNA repair system in the cell mainly includes five pathways: direct damage reversal repair, base excision repair, nucleotide excision repair, recombination repair, and mismatch repair (13). If the repair function is defective, or when a key protein in a specific DNA damage repair pathway is mutated, DNA damage may lead to two results: one is cell death; the other is gene mutation, or malignant transformation into tumor cells. It is worth noting that although defects in DNA repair function can cause tumors, the DNA repair function of cancer cells is not reduced; on the contrary, it is significantly increased, and can fully repair the DNA damage caused by chemotherapeutic drugs. This is also one of the reasons why most anti-cancer drugs are not effective (14).

Therefore, in this study the combined action of ROS genes with DNA repair genes on the prognosis of patients diagnosed with lung adenocarcinoma (LUAD) was explored. Since this is a cancer type with a high incidence and high mortality rate, our aim was to find a potential correlation between ROS genes and DNA repair genes, to evaluate whether the inhibition of the repair of damaged tumor cells could increase tumor cell death and ameliorate the prognosis of patients. In this way, a potential combined therapeutic therapy can be also considered.

## Materials and Methods

### Data Source and Pre-processing

The RNA-Seq based transcriptome profiles (FPKM; Fragments Per Kilobase of transcript per Million mapped reads) and corresponding clinical data of LUAD patients were downloaded from the Cancer Genome Atlas (TCGA) portal using the gdc-client software downloading tool. Additionally, the gene expression profiles in LUAD patients (GSE68465, sequenced using Affymetrix, HG-U133A plus 2.0 Array, up to November 2020) were also obtained from the Gene Expression Omnibus (GEO) database (http://www.ncbi.nlm.nih.gov/geo/). All analyses were performed using the R software (R Foundation for Statistical Computing, Vienna, Austria, 3.4.1 Version).

### ROS and DNA Repair Gene Acquisition and Sorting

The ROS-related genes and DNA repair genes were downloaded from the Molecular Signatures Database (MSigDB) for use with the Gene Set Enrichment Analysis (GSEA) database. The intersection of these genes with the genes from TCGA was used to obtain the final ROS-related genes and DNA repair genes. The TCGA samples with incomplete clinical data and survival time <30 days were not taken into consideration and consequently removed.

### Consistent Clustering and Screening of ROS-Related Genes and DNA Repair Related Genes

The ConsensusClusterPlus package of R was used to cluster ROS-related genes and DNA repair genes separately, and the survival analysis was performed to compare the prognostic differences of different categories. Genes showing significant differences in their expression in tumor samples and normal samples were obtained, the screening conditions were set at *p* < 0.05 and |LogFC|>1, and finally the expression of differential genes in different categories were analyzed according to ROS genes and DNA repair genes.

### Sample Reclassification and Differential Gene Expression Analysis in Different Prognostic Categories

The categories and prognosis of some samples of the two clustering methods were different. The samples obtained from the two clusters are reclassified in an interactive manner and called ROS_Cn_DNA_Repair_Cm (Table 1). Then, differential genes were compared in different categories according to ROS genes and DNA repair genes in the new category.

**Table 1:** Reclassified samples correspond to samples independently classified based on ROS genes and DNA repair genes.

**ROS_cluster**	**DNA_Repair_cluster**	**Subtype**
ROS_C1	DNA_Repair_C1	ROS_C1_DNA_Repair_C1
ROS_C1	DNA_Repair_C3	ROS_C1_DNA_Repair_C3
ROS_C2	DNA_Repair_C5	ROS_C2_DNA_Repair_C5
ROS_C2	DNA_Repair_C6	ROS_C2_DNA_Repair_C6
ROS_C3	DNA_Repair_C1	ROS_C3_DNA_Repair_C1
ROS_C3	DNA_Repair_C3	ROS_C3_DNA_Repair_C3
ROS_C4	DNA_Repair_C1	ROS_C4_DNA_Repair_C1
ROS_C4	DNA_Repair_C4	ROS_C4_DNA_Repair_C4
ROS_C4	DNA_Repair_C5	ROS_C4_DNA_Repair_C5
ROS_C5	DNA_Repair_C2	ROS_C5_DNA_Repair_C2

### Regulatory Network and Correlation Analysis Among Target Genes

ROS-related and DNA repair genes significantly different in the new categories were obtained where the samples obtained from the two clusters are reclassified in an interactive manner and called ROS_Cn_DNA_Repair_Cm. A regulatory network was constructed using the STRING database, the correlation coefficient between the two set of genes at the same time was calculated, and then the relationship between ROS-related and DNA repair genes was obtained.

### LASSO Regression Analysis for the Construction of the Prognostic Gene Model

Univariate Cox proportional hazards regression analysis was performed to screen target ROS-related genes and DNA repair genes significantly associated with overall survival (OS) in the TCGA LUAD dataset. Then, LASSO Cox regression analysis of the identified OS-related genes was performed using the R-glmnet package. Multivariable Cox proportional hazards regression analysis was performed to establish the prognostic model of the target genes. The LUAD samples were divided into high risk and low risk by the median risk score; the Kaplan–Meier curve was constructed, and the log-rank test was conducted to compare the survival differences between the two groups. The ROC curve was used to evaluate the accuracy of the model. GSE68465 data were used as the validation set to further confirm the model.

## Results

### Data Processing Results

The ROS-related gene set as the hallmark of ROS-related pathway containing 49 genes, and the DNA repair gene set Kauffmann DNA repair genes (1) containing 230 DNA repair genes were downloaded from the MSigDB and used with the GSEA. The intersection of these genes with the genes from TCGA resulted in a total of 45 ROS-related genes and 194 DNA repair genes. The TCGA samples with incomplete clinical data and survival time <30 days were not taken into consideration and removed, and the data of 465 samples were collected for further analysis.

### Consistent Clustering and Screening of ROS-Related Genes and DNA Repair Genes

**Figure 1:**
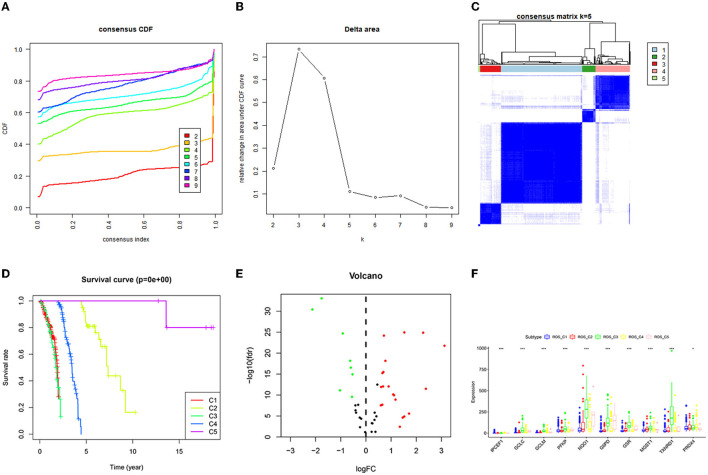
Consistent clustering results of ROS-related genes and screening of differential genes. **(A)** Consistent Cumulative Distribution Function (CDF) diagram: this diagram shows the cumulative distribution function when k takes different values, which is used to determine when k takes the value, CDF reaches an approximate maximum value, and the cluster analysis result is the most reliable at this time. **(B)** Delta Area Plot: this graph shows the relative change of the area under the CDF curve between k and k-1. When k = 6, the area under the curve only increases slightly, so 5 is the appropriate value of k. **(C)** Matrix heat map when k = 5: the rows and columns of the matrix are all samples, and the values of the consistency matrix range from 0 (it is impossible to cluster together) to 1 (always cluster together) from white to dark blue Color indicates that the consistency matrix is arranged according to the consistency classification (the tree diagram above the heat map). The bar between the dendrogram and the heat map is the category. **(D)** Survival prognosis curves of different categories. **(E)** The differential gene volcano map describes the situation of the differential gene. The y-axis of the volcano graph is -log10 (Q-value), that is, qvalue (value after *p*-value correction) is –log10, so the higher the value, the smaller the qvalue is, the more significant it is. The abscissa is Log2 fold change, that is, log2 is taken for fold change, so the closer the points on both sides (each point represents a gene), the greater the increase or decrease in gene expression. **(F)** Genes with significant differences in the C1–C5 categories.

The consistent clustering of TCGA_ROS data divided the 465 samples into five categories. The survival analysis of the 5 categories revealed a significant difference in survival, with the category C3 having the worst prognosis, while the C5 having the best prognosis. The difference analysis resulted in a total of 14 ROS-related genes (11 up-regulated and 3 down-regulated genes). Then, the expression of differential genes in the 5 categories was compared, and 10 genes were significantly different in C1–C5 (Figure 1).

**Figure 2:**
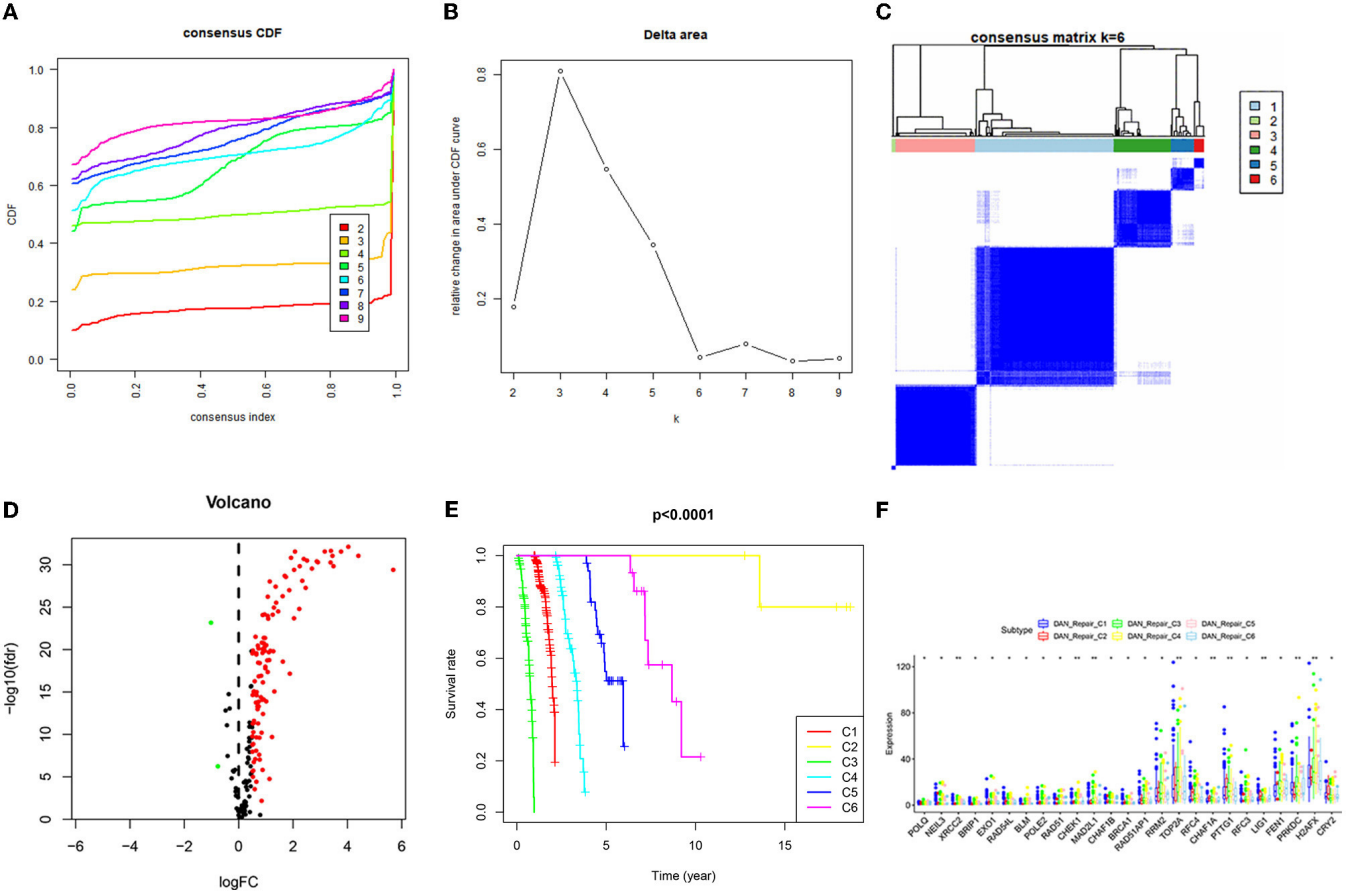
Consistent clustering results of DNA repair related genes and screening of differential genes. **(A)** Consistent Cumulative Distribution Function (CDF) diagram: this diagram shows the cumulative distribution function when k takes different values, which is used to determine when k takes the value, CDF reaches an approximate maximum value, and the cluster analysis result is the most reliable at this time. **(B)** Delta Area Plot: this graph shows the relative change of the area under the CDF curve between k and k-1. When k = 7, the area under the curve only increases slightly, so 6 is the appropriate value of k. **(C)** Matrix heat map when k = 6: the rows and columns of the matrix are all samples, and the values of the consistency matrix range from 0 (it is impossible to cluster together) to 1 (always cluster together) from white to dark blue Color indicates that the consistency matrix is arranged according to the consistency classification (the tree diagram above the heat map). The bar between the dendrogram and the heat map is the category. **(D)** The differential gene volcano map describes the situation of the differential gene. The y-axis of the volcano graph is –log10 (Qvalue), that is, qvalue (value after pvalue correction) is –log10, so the higher the value, the smaller the qvalue is, the more significant it is. The abscissa is Log2 fold change, that is, log2 is taken for fold change, so the closer the points on both sides (each point represents a gene), the greater the increase or decrease in gene expression. **(E)** Survival prognosis curves of different categories. **(F)** Genes with significant differences in the C1–C6 categories.

Similar to the above procedure, the consistent clustering of TCGA_DNA repair gene data divided the 465 samples into 6 categories, and survival analysis of these 6 categories revealed that C3 had the worst prognosis, while C2 had the best prognosis. Forty-nine DNA-related differential genes (48 up-regulated genes and 1 down-regulated gene) were obtained, the differences of genes in the 6 categories were compared, and the results revealed that 25 genes were significantly different in C1–C6 (Figure 2).

**Figure 3:**
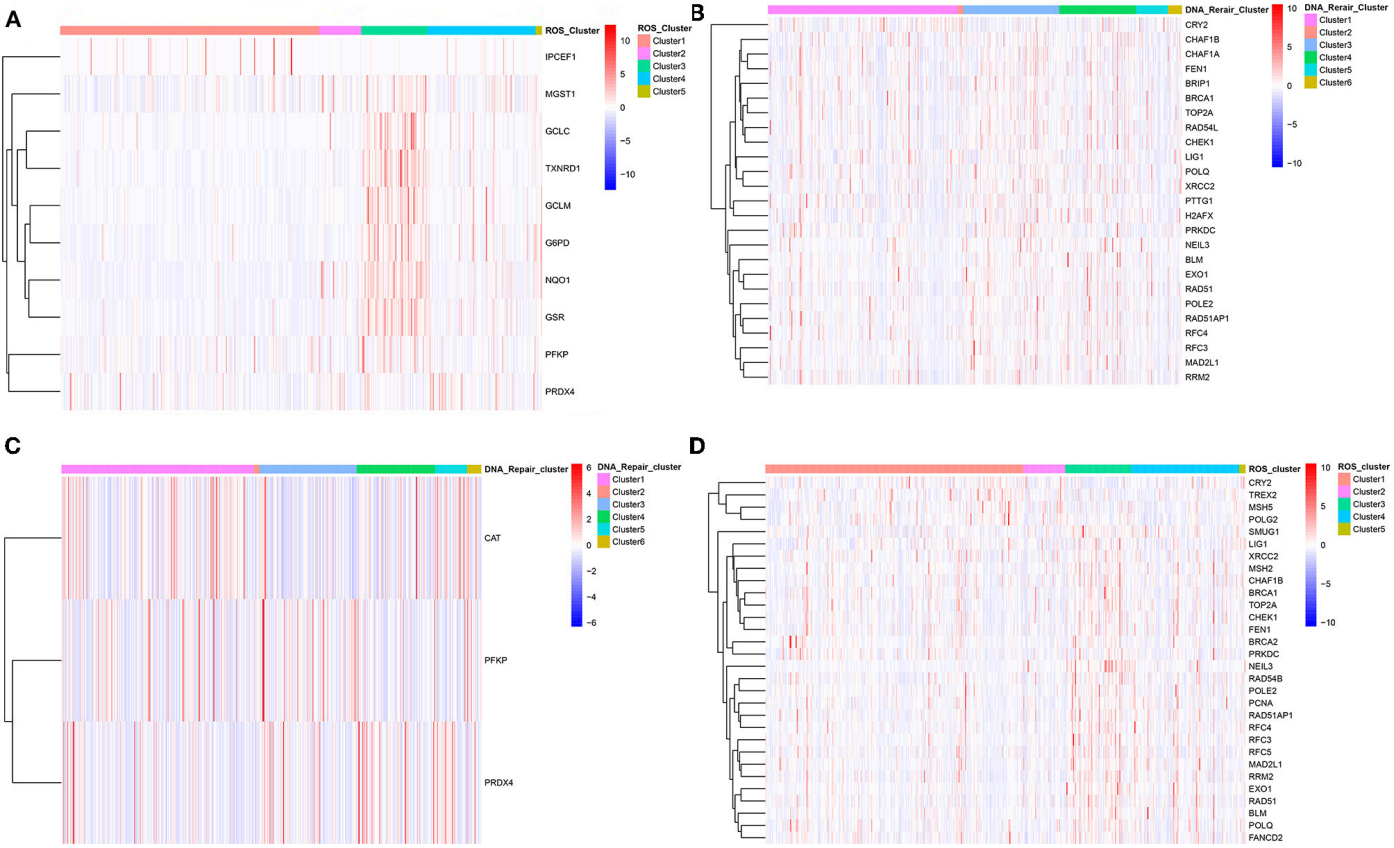
Clustering heat map of ROS-related differential genes and DNA repair-related differential genes in different categories. **(A)** Clustering heat map of differential genes in C1–C5 categories of ROS clustering. **(B)** Clustering heat map of DNA repair-related differential genes in C1–C6 categories of DNA-repair clustering. **(C)** Clustering heat map of ROS-related differential genes in C1–C6 categories of DNA-repair clustering. **(D)** Clustering heat map of DNA-repair-related differential genes in C1–C5 categories of ROS clustering.

Subsequently, ROS-related and DNA repair genes were visualized in the ROS classification and DNA repair genes and ROS-related genes were visualized in the DNA classification in order to observe the overall expression of genes in the two classifications. Certain differences in the expression of ROS-related and DNA repair genes existed, corresponding to different clustering methods. The most intuitive reaction was that ROS_C3 had the most different prognosis, and the ROS-related and DNA repair genes contained in it were highly expressed. The differences in the expression of the two categories of genes in other categories were not the same, which might be related to the mutual regulation of the two categories of genes (Figure 3).

### Differences in Survival and Gene Expression in the Reclassification Samples

**Figure 4:**
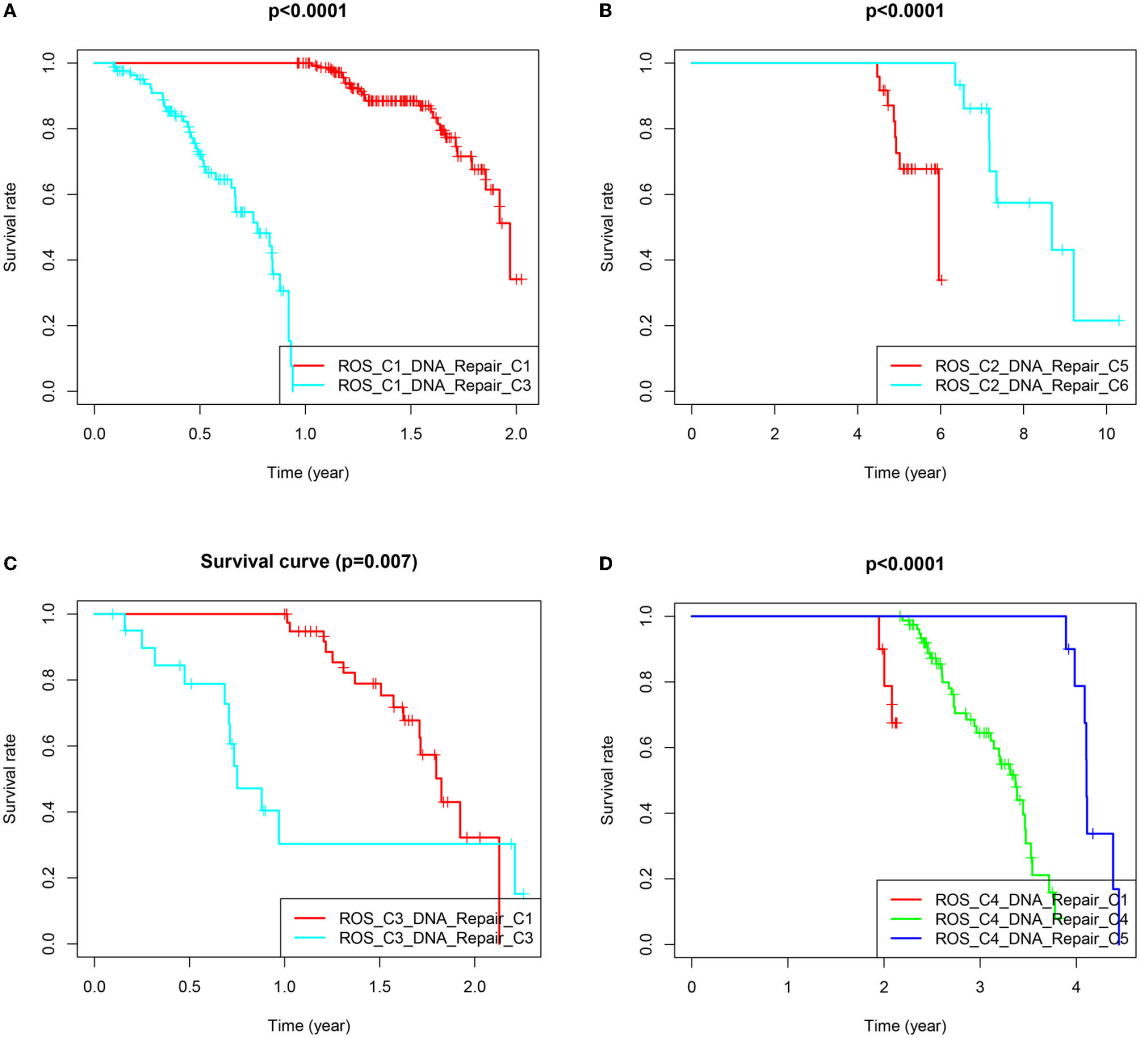
Survival prognostic curves of reclassified samples in different classifications. **(A)** Survival prognostic curves of ROS_C1_DNA_Repair_C1 and ROS_C1_DNA_Repair_C3. **(B)** Survival prognostic curves of ROS_C2_DNA_Repair_C5 and ROS_C2_DNA_Repair_C6. **(C)** Survival prognostic curves of ROS_C3_DNA_Repair_C1 and ROS_C3_DNA_Repair_C3. **(D)** Survival prognostic curves of ROS_C4_DNA_Repair_C1, ROS_C4_DNA_Repair_C4 and ROS_C4_DNA_Repair_C5.

**Figure 5:**
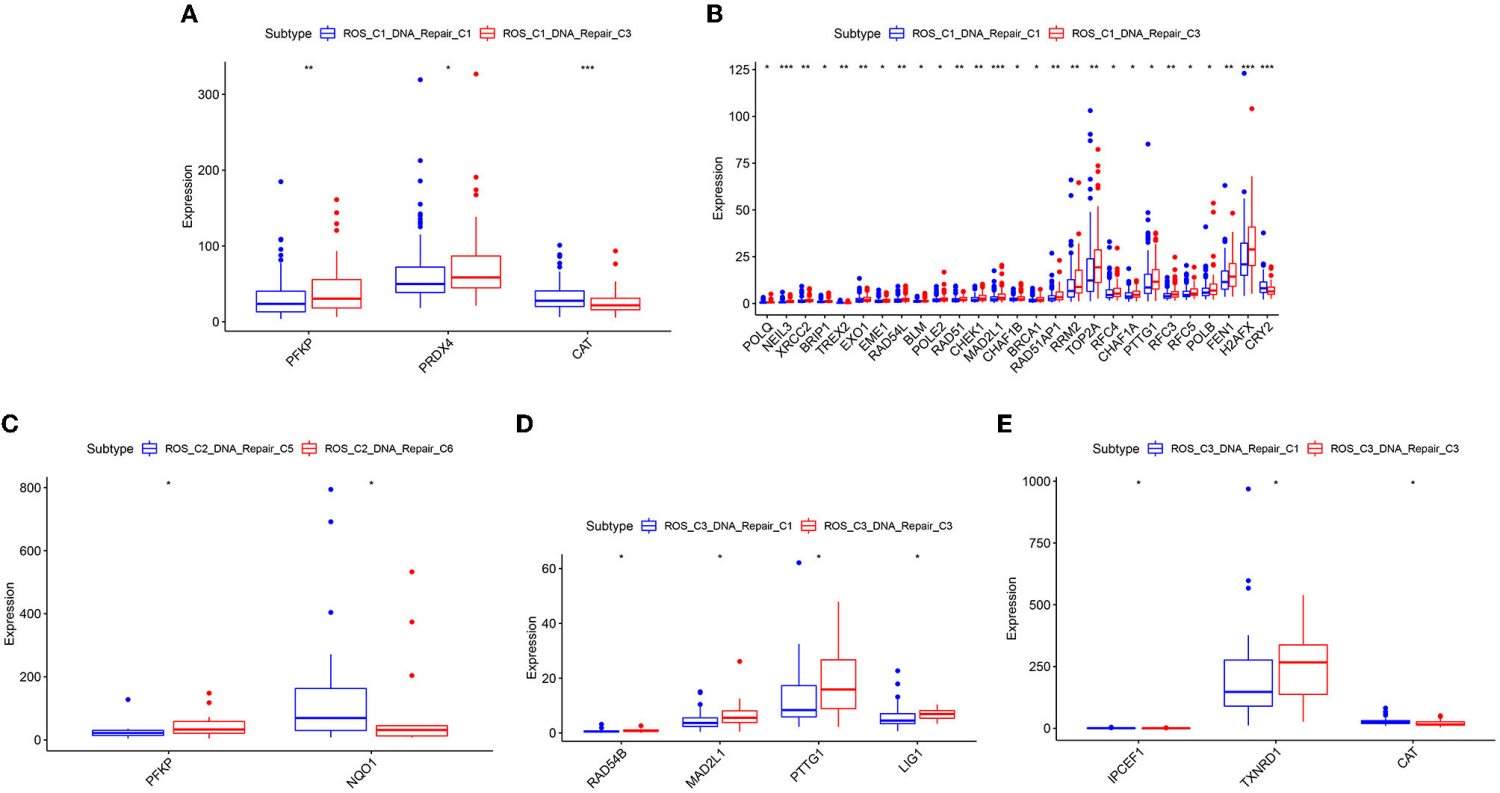
Differentially expressed genes of reclassified samples in different classifications. **(A)** ROS-related differential genes with obvious differences between ROS_C1_DNA_Repair_C1 and ROS_C1_DNA_Repair_C3. **(B)** DNA repair-related differential genes with obvious differences between ROS_C1_DNA_Repair_C1 and ROS_C1_DNA_Repair_C3. **(C)** ROS-related differential genes with obvious differences between ROS_C2_DNA_Repair_C5 and ROS_C2_DNA_Repair_C6. **(D)** DNA repair-related differential genes with obvious differences between ROS_C3_DNA_Repair_C1 and ROS_C3_DNA_Repair_C3. **(E)** ROS-related differential genes with obvious differences between ROS_C3_DNA_Repair_C1 and ROS_C3_DNA_Repair_C3.

The samples obtained from the two clusters were interactively divided into ten categories, as shown in Table 1. The survival analysis revealed that the survival prognosis of the patients whose samples that originally belonged to the ROS category was significantly different after regrouping. The comparison of the expression of the genes between the different new classifications that originally belonged to the ROS category revealed that the higher the expression of up-regulated ROS-related and DNA repair genes, the worse the prognosis, while the down-regulated genes (CYR2, PFKP, CAT) were positively correlated with a longer survival (Figures 4, 5; Table 2).

**Table 2:** Up-regulated and down-regulated genes related to the prognosis of reclassified samples.

**Subtype**	**Survival prognosis**	
	**Bad**	**Good**
	**Up regulated genes**	**Down regulated genes**
ROS_C1_DNA_Repair_C1 ROS_C1_DNA_Repair_C3	PRDX4 POLQ NEIL3 XRCC2 BRIP1 TREX2 EXO1 EME1 RAD54L BLM POLE2 RAD51 CHEK1 MAD2L1 CHAF1B BRCA1 RAD51AP1 RRM2 TOP2A RFC4 CHAF1A PTTG1 RFC3 RFC5 POLB FEN1 H2AFX	PFKP CAT CYR2
ROS_C2_DNA_Repair_C5 ROS_C2_DNA_Repair_C6	NQO1	PFKP
ROS_C3_DNA_Repair_C1 ROS_C3_DNA_Repair_C3	LIG1 MAD2L1 PTTG1 RAD54B IPCEF1 TXNRD1	CAT

### Regulatory Network and Correlation Analysis Among Target Genes

**Figure 6:**
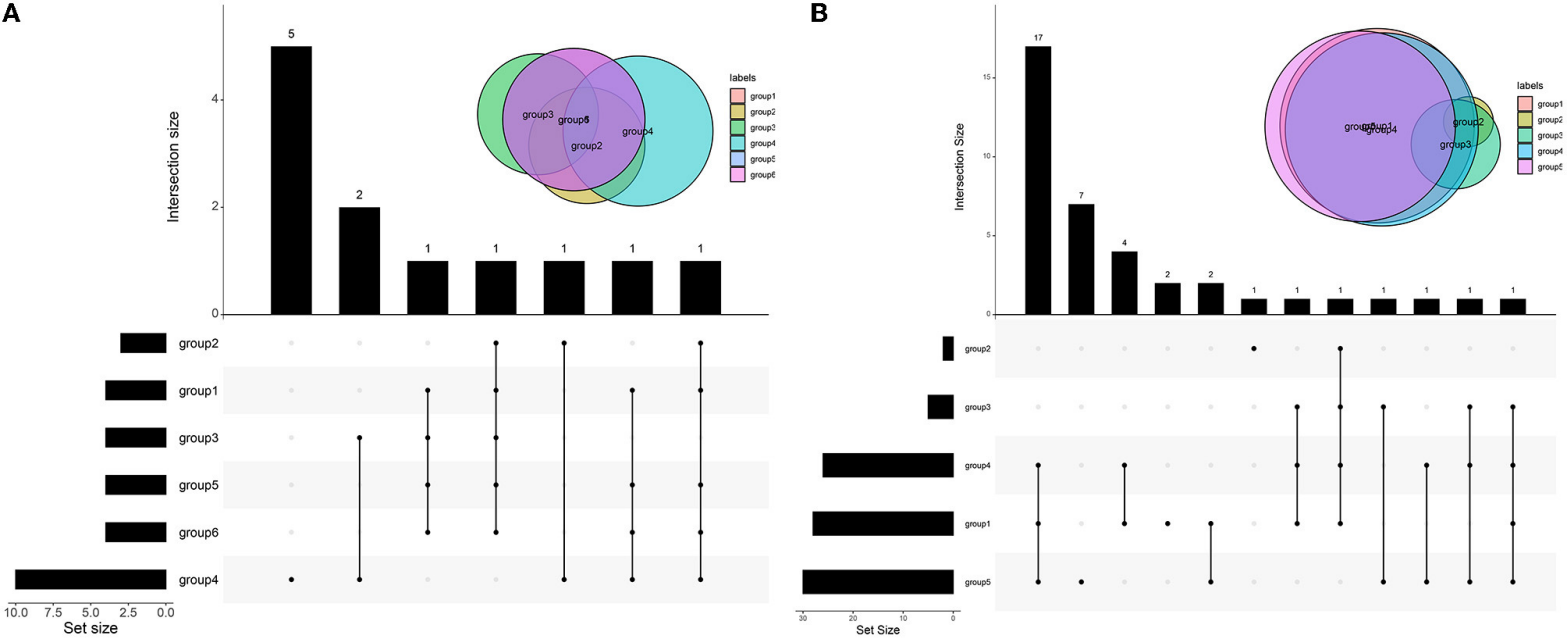
Venn diagram of differential genes in different categories. **(A)** Venn diagrams of ROS-related differential genes in different categories. **(B)** Venn diagrams of DNA repair-related differential genes in different categories. The black in the figure indicates that there is data at that location, and the gray point indicates that there is no data. Connecting different points indicates that there is an intersection. See the bar chart above for specific data. See the bar graph on the left for the total amount of different types of data.

**Figure 7:**
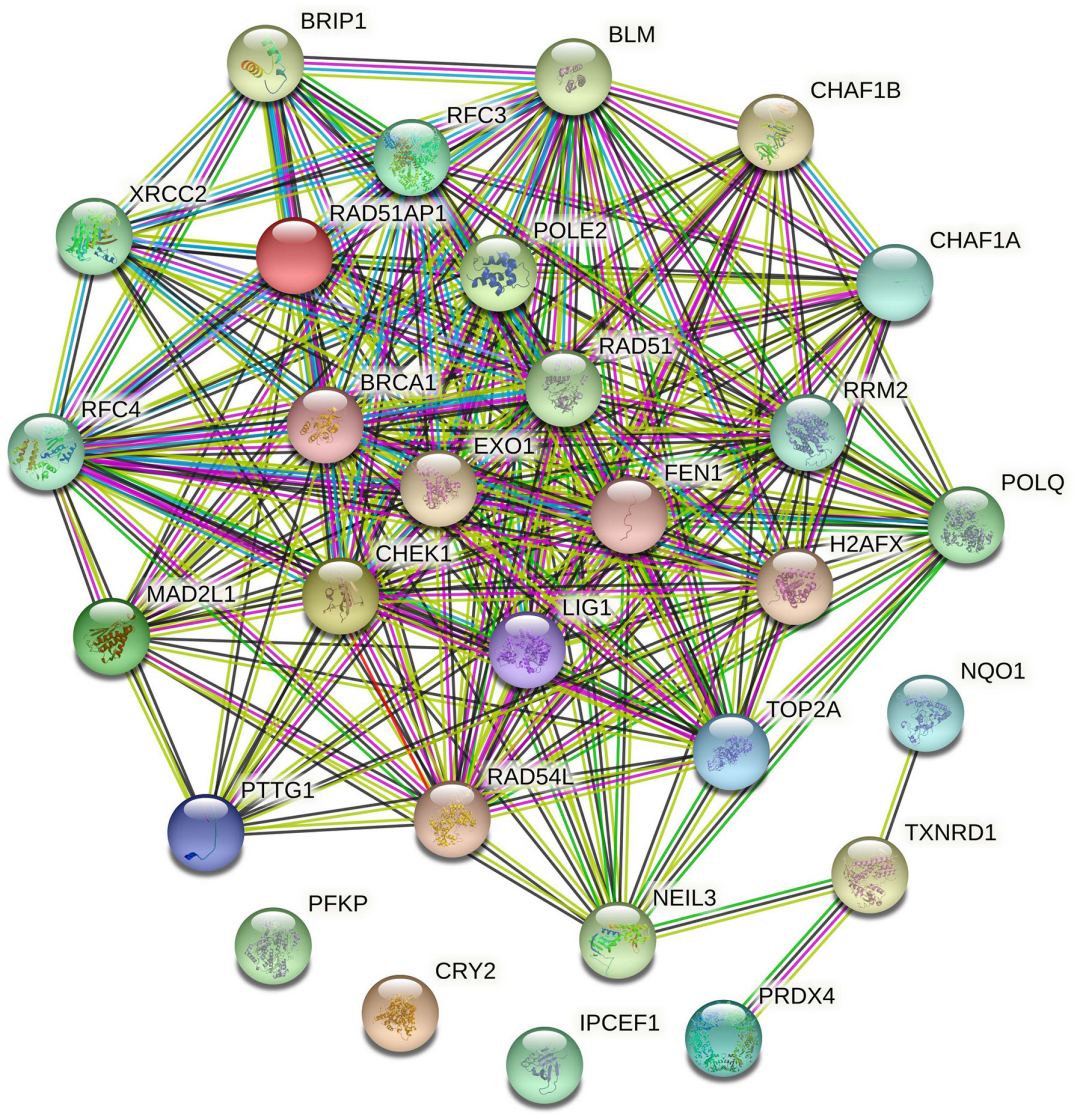
Regulatory network of ROS-related genes and DNA repair genes. The ROS-related genes NQO1, TXNRD1, and PRDX4 could establish links with other DNA repair genes through the DNA repair gene NEIL3.

**Figure 8:**
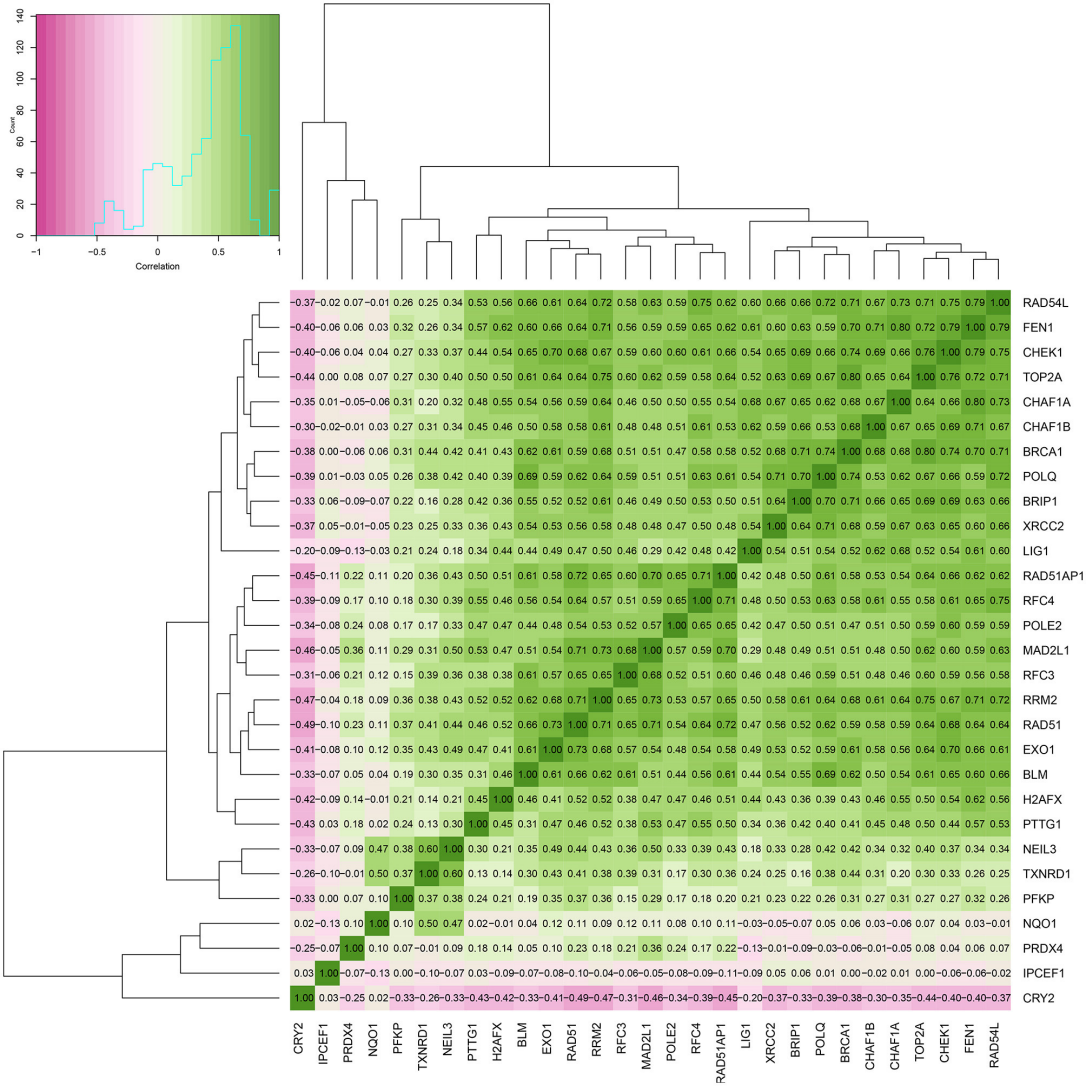
Heat map of the correlation between ROS-related genes and DNA repair genes.

**Figure 9:**
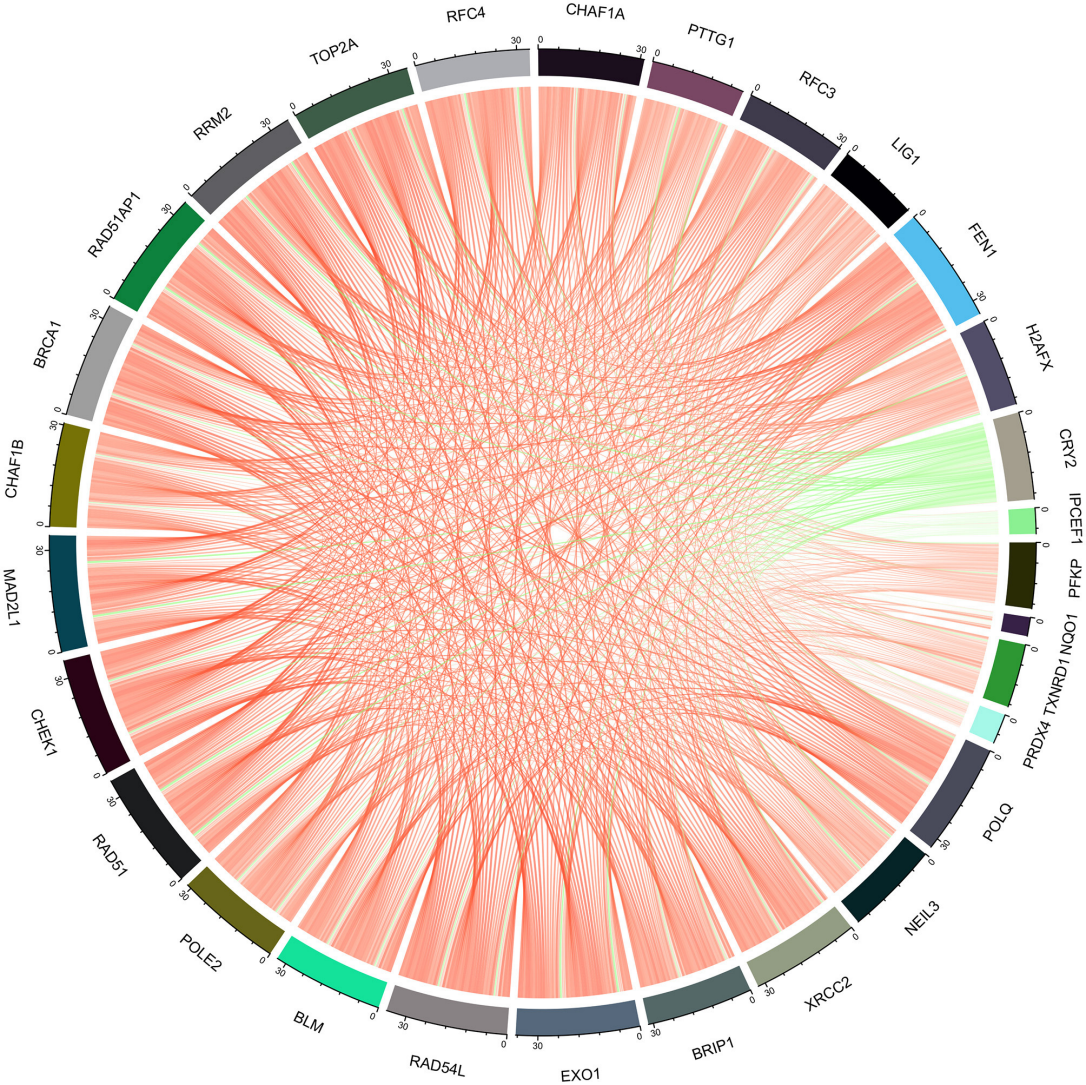
Chord diagram of the correlation between ROS-related genes and DNA repair genes.

The enrichment of differential ROS-related and DNA repair genes in the ROS_Cn_DNA_Repair_Cm category was visualized by the Venn diagram, and the intersection between the differential genes of the ROS and DNA repair categories was performed to obtain a total of 29 target genes (Figure 6). These 29 differentially enriched genes were imported into STRING to construct a gene regulation network and calculate the correlation coefficient among genes. The results showed that the DNA repair genes had a strong internal regulatory relationship. DNA repair and ROS-related genes could be linked through NEIL3-TXNRD1, and the Pearson correlation coefficient between the two was 0.60. In addition, the CYR2 gene showed a negative correlation with other ROS-related and DNA repair genes, while NQO1, PRDX4, and IPCEF1 showed a weak negative correlation with other genes (Figures 7–9).

### Prognostic Model and Genes Associated With Prognosis

**Figure 10:**
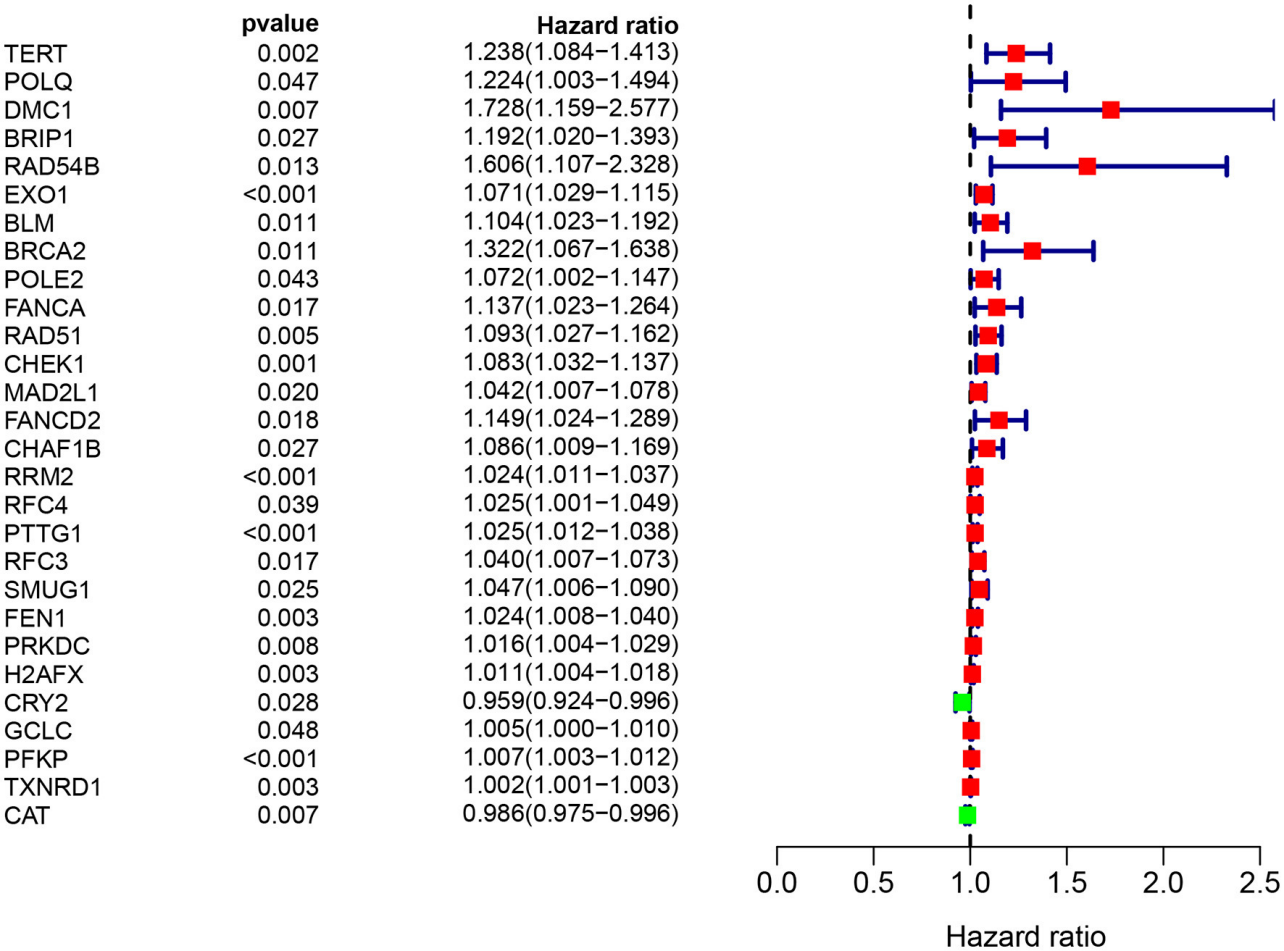
Target genes screened by univariate prognostic analysis.

**Figure 11:**
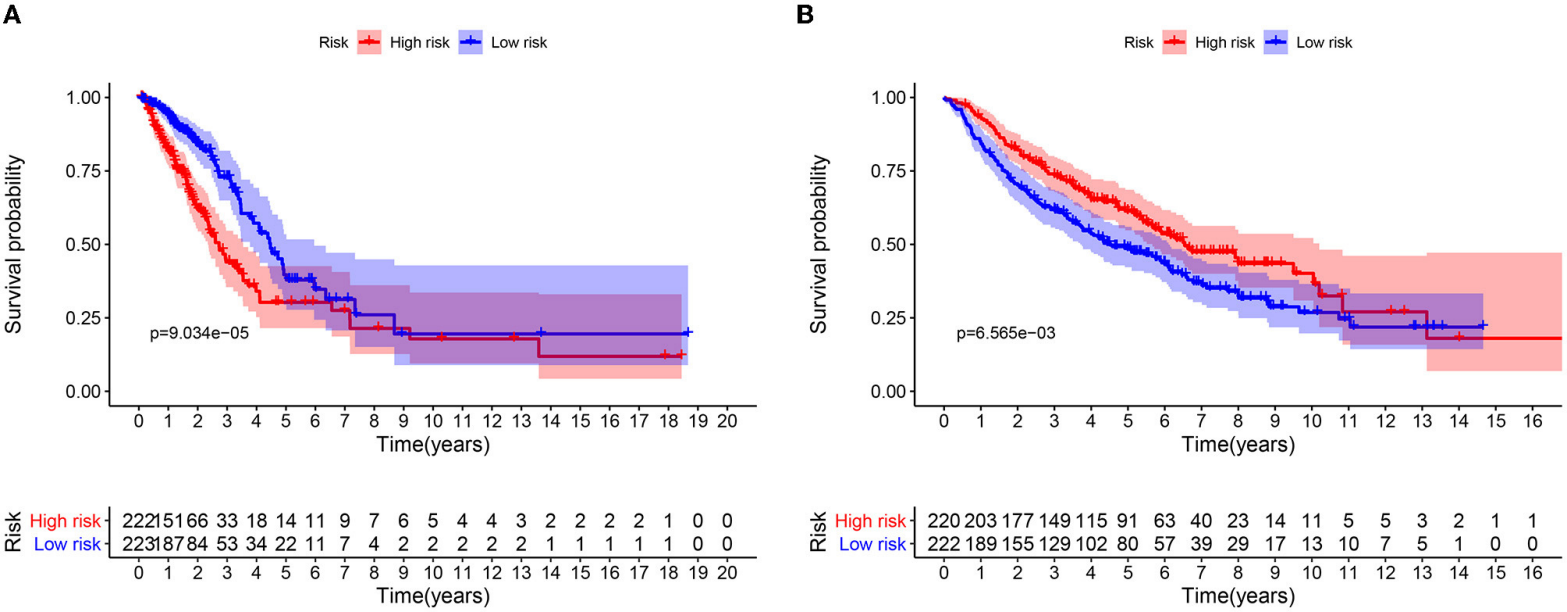
Kaplan-Meier analysis of OS for LUAD patients using TCGA and GEO database. **(A)** Kaplan-Meier survival curves of the relative OS of high- and low-risk groups in TCGA database. **(B)** Kaplan-Meier survival curves of the relative OS of high- and low-risk groups in GEO database.

**Figure 12:**
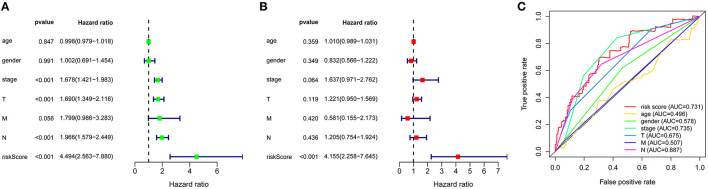
Construction of ROS and DNA-repair-related genes model for patients with LUAD. **(A)** Prognostic values of ROS and DNA-repair -related genes by univariate Cox regression analysis. **(B)** Prognostic values of ROS and DNA-repair -related genes by multivariate Cox regression analysis. **(C)** ROC curve of ROS and DNA-repair -related genes.

A total of 49 DNA repair and 14 ROS-related genes from the TCGA LUAD data were analyzed by Univariate Cox regression. Twenty-eight genes were associated with a prognosis and were entered into the LASSO regression analysis (Figure 10), and a total of eight genes (TERT, PTTG1, SMUG1, PRKDC, H2AFX, PFKP, TXNRD1, and CAT) were identified to build the model. The prognostic value of the risk scores was assessed, which were estimated with the formula: risk score = ∑ Xβ^*^ coef β, where coef β was the coefficient and Xβ was the gene relative expression (risk score = TERT^*^0.102+PTTG1^*^0.012+SMUG1^*^0.123+PRKDC^*^ 0.005+ H2AFX^*^0.002+ PFKP^*^0.003+TXNRD1^*^0.0006+CAT^*^-0.003). As regard the TCGA LUAD data, the risk score in both univariate and multivariate analysis was significantly related to OS (HR = 4.494, 95% CI = 2.563–7.880, *p* < 0.001; HR =4.155, 95% CI = 2.258–6.645, *p* < 0.001, respectively) (Figures 12A,B). The patients with low-risk scores showed a significantly better prognosis than those with a high-risk score (Figures 11A,B) both in TCGA and GEO LUAD data, as demonstrated by the Kaplan–Meier cumulative curve. The AUC of the risk score was 0.731, which implied that the Cox model could predict the prognosis quite well (Figure 12C).

## Discussion

ROS is produced in many cellular compartments including mitochondria, which are the major source of ROS (mROS) (15). Superoxide anion (∙O2-), hydrogen peroxide (H2O2) and hydroxyl radical (∙OH) belong to a group of highly reactive and heterogeneous molecules derived from oxygen (O2) and are the main forms of ROS in biological systems (16). Many factors in the tumor microenvironment, including the presence of ROS, promote the progress of solid tumors. The increase of ROS level, the imbalance of redox homeostasis and the enhancement of antioxidant capacity are some of the many signs in cancer cells. Therefore, the understanding and elucidating the role of ROS in the tumor microenvironment is essential for developing new methods to combat this disease (17). Various tumors, including LUAD, possess high levels of ROS with abnormal metabolism and constitutive carcinogenic signals. ROS are the main effectors of DNA damage associated with cancer and is accompanied by tumor suppression (18, 19). Therefore, tumor cells adapt to the oxidative DNA damage to prevent cell destruction by regulating cell necrosis through the modification in the expression of some genes, thereby inducing the aberrant expression of signaling networks that cause tumorigenesis and metastasis (20). 8-hydroxyguanine is the strongest product of oxidative stress in cells, and is mostly closely related to the occurrence and development of tumors. The DNA repair gene can hydrolyze 8-hydroxyguanine in the base pool to avoid base mismatch and replacement. Once the 8-hydroxyguanine in tumor cells is hydrolyzed by the DNA repair gene, it promotes tumor cell growth. Certain protective effects lead to a malignant phenotype, poor cancer prognosis, or resistance to treatment (21, 22). In some cases, tumors up-regulate the mutagenic repair pathways to survive. Therefore, cancer cells generally rely more on repair pathways than normal cells. In addition, cancer cells often have dysfunctional redox homeostasis, and therefore once again, they rely heavily on mechanisms that repair oxidative DNA damage and inhibit enzymes that modify compounds, which can then be incorporated into genomic DNA in their unmodified form. Processes such as replication and oxidative stress provide a background for ongoing DNA damage in cancer cells and can provide a potential therapeutic window for compounds that exacerbate these processes. Such compounds can accomplish by further emphasizing replication, weakening the ability of cancer cells to handle high levels of replication or oxidative stress, or potentially inhibiting DNA repair and related processes (23–25).

Therefore, in this work, the synergistic tumorigenic effect of ROS-related genes and DNA repair genes was evaluated, and the regulatory relationship between the two groups of genes was further explored. It is important to consider whether it is better to use ROS to kill cancer cells or to inhibit the DNA repair in cancer cells to improve patient prognosis.

The expression of ROS-related genes and DNA repair genes was used to cluster TCGA tumor samples uniformly. ROS-related genes divided tumors into classes, and DNA repair genes divided tumor samples into classes. Significant differences in survival between the internal classifications were obtained by the two clustering methods, and the differentially expressed genes were further screened. Our analysis found that the samples that originally belonged to the ROS classification partial overlapped in the classification of DNA repair genes. After reclassifying the samples according to the two classifications, the prognosis of patients changed when the expression of ROS-related and DNA repair genes in the samples changed. Thus, our hypothesis was that ROS-related and DNA repair genes might have a mutual regulatory relationship, which in turn affected the occurrence and development of tumors. A total of 29 differential genes were finally identified and included 5 ROS-related and 24 DNA repair genes. STRING analysis of the regulatory relationship found that 3 ROS-related genes (NQO1, TXNRD1, and PRDX4) can be repaired by the DNA repair gene NEIL3 and other DNA repair genes.A large amount of evidence showed that NQO1 has a “Janus” effect in cancer biology, playing a role in suppressing cancer and promoting tumors (26). NQO1 is constitutively expressed at a relatively low level in various normal tissues. Under oxidative stress, NF-E2 p45-related factor 2 (Nrf2)/Kelch-like ECH-related protein 1 (Keap1) signaling pathway can cooperate to transcribe a series of defense genes and provide cells with multiple layers of protection against carcinogenesis. These measures include the immediate elimination of ROS (27). The expression of NQO1 is considered as a practical and economical way to control cancer. NQO1 is abnormally up-regulated in solid tumors, and high levels of NQO1 are associated with poor patient prognosis. It is known that cancer cells have a significant increase in ROS production compared to normal cells. In this case, high levels of NQO1 in cancer can help cancer cells to cope with the increased ROS just like normal cells, thus, tumor growth and metastasis is not only not compromised, but promoted (28). Our results showed that NQO1 was correlated with the expression of the DNA repair gene NEIL3 (Pearson correlation coefficient), suggesting its role as a tumor control gene.

The cytoplasmic selenoprotein thioredoxin reductase 1 (TXNRD1) has several different effects related to cancer including the protection of normal cells to evolve into cancer cells or the protection against the promotion of cancer progression. TXNRD1 has a unique connection with Nrf2 signaling and ribonucleotide reductase-dependent deoxyribonucleotide production and it supports a variety of antioxidant systems against oxidative stress. Thus, it is essential that metabolic pathways regulated by TrxR1 are affected in cancer (29). Our regulatory network suggested that TXNRD1 had a significant correlation with the DNA repair gene NEIL3, thus, it might be considered as a potential targeted gene in a combination therapy affecting ROS-related genes and DNA repair genes.

Peroxiredoxin 4 is a typical peroxidase 2-Cys antioxidant in the endoplasmic reticulum, which protect cells against oxidative stress by detoxifying hydrogen peroxide, thus promoting cell survival (30). The role of PRDX4 in cancer received considerable attention. The expression of PRDX4 in NSCLC-derived endothelial cells is higher than that in normal cells (31). Sulfiredoxin is an antioxidant protein induced by H2O2 that acts as a catalyst for reducing the peroxidized PRDXs to reduce their peroxidase activity. Sulfiredoxin is more inclined to combine with PRDX4 than other PRDXs. The up-regulation or down-regulation of the sulfiredoxin-PRDX4 axis can affect the mitogen-activated protein kinase pathway, cAMP response element binding protein and activator protein-1/matrix metalloproteinase axis pathway (32). Furthermore, another study revealed that the expression of PRDX4 is closely related to the disease-free survival time and short recurrence time of patients with early-stage lung squamous cell carcinoma undergoing early radical surgery (33).

Endonuclease VIII-like 3 (NEIL3) is a DNA glycosylase protein that is involved in oxidative and interstrand crosslink DNA damage repair (34). NEIL3 is highly expressed in various human cancer cells and is associated with metastatic cancer, indicating that it may be necessary to maintain cancer cell growth or malignant progression (21, 35). NEIL3 overexpression is positively correlated with homologous recombination and mismatch repair gene expression. High NEIL3 expression may promote cancer phenotype by increasing genomic instability and/or interfering with other DNA repair (34). Our analysis found that NEIL3 played a pivotal role in the connection between DNA repair genes and ROS-related genes. Therefore, the mutual regulation of ROS-related and DNA repair genes centered on NEIL3 might become an important topic for further studies.

A prognostic model based on all differentially expressed ROS-related genes and DNA repair genes was constructed and combined with the clinical data of the samples, and finally nine genes were selected to calculate the risk score. The results revealed that the prognosis of patients in the high- and low-risk groups was significantly different, and the GEO data verified this result. The multivariate analysis suggested that the risk score could be used as an independent prognostic factor to evaluate patient prognosis. The above mentioned model genes included three ROS-related genes and six DNA repair genes, and TXNRD1 gene played an important role in the regulatory network of the two groups of genes, as revealed by previous studies.

## Conclusion

This study might highlight the significance of ROS-related and DNA repair genes in LUAD, and the combined target of ROS and DNA repair genes might be a promising strategy in the treatment of LUAD, although further studies should be performed to validate these findings.

## Data Availability Statement

The datasets presented in this study can be found in online repositories. The names of the repository/repositories and accession number(s) can be found in the article/supplementary material.

## Author Contributions

YQ has designed the research. H-MF and YZ analyzed data and wrote the paper. H-MF retrieved and collected data. W-JY were responsible for drawing. All authors have read and approved the final manuscript.

## Conflict of Interest

The authors declare that the research was conducted in the absence of any commercial or financial relationships that could be construed as a potential conflict of interest.

## Publisher's Note

All claims expressed in this article are solely those of the authors and do not necessarily represent those of their affiliated organizations, or those of the publisher, the editors and the reviewers. Any product that may be evaluated in this article, or claim that may be made by its manufacturer, is not guaranteed or endorsed by the publisher.
